# Computer-Aided Prediction of Long-Term Prognosis of Patients with Ulcerative Colitis after Cytoapheresis Therapy

**DOI:** 10.1371/journal.pone.0131197

**Published:** 2015-06-25

**Authors:** Tetsuro Takayama, Susumu Okamoto, Tadakazu Hisamatsu, Makoto Naganuma, Katsuyoshi Matsuoka, Shinta Mizuno, Rieko Bessho, Toshifumi Hibi, Takanori Kanai

**Affiliations:** 1 Division of Gastroenterology and Hepatology, Department of Internal Medicine, Keio University School of Medicine, Shinjuku-ku, Tokyo, Japan; 2 Predictive Medicine Institute, Minato-ku, Tokyo, Japan; 3 Tokai University School of Medicine, Isehara-shi, Kanagawa, Japan; 4 Wata Clinic, Katsushika-ku, Tokyo, Japan; 5 Department of General Medicine, Kyorin University School of Medicine, Mitaka-shi, Tokyo, Japan; 6 Center for Diagnostic and Therapeutic Endoscopy, Keio University School of Medicine, Shinjuku-ku, Tokyo, Japan; 7 Department of Advanced Therapeutics for Gastrointestinal Diseases, Graduate School, Tokyo Medical and Dental University, Bunkyo-ku, Tokyo, Japan; 8 Department of Internal Medicine, Saiseikai Central Hospital, Minato-ku, Tokyo, Japan; 9 Center for Advanced IBD Research and Treatment, Kitasato Institute Hospital, Kitasato University,Minato-ku, Tokyo, Japan; Massachusetts General Hospital, UNITED STATES

## Abstract

Cytoapheresis (CAP) therapy is widely used in ulcerative colitis (UC) patients with moderate to severe activity in Japan. The aim of this study is to predict the need of operation after CAP therapy of UC patients on an individual level using an artificial neural network system (ANN). Ninety UC patients with moderate to severe activity were treated with CAP. Data on the patients’ demographics, medication, clinical activity index (CAI) and efficacy of CAP were collected. Clinical data were divided into training data group and validation data group and analyzed using ANN to predict individual outcomes. The sensitivity and specificity of predictive expression by ANN were 0.96 and 0.97, respectively. Events of admission, operation, and use of immunomodulator, and efficacy of CAP were significantly correlated to the outcome. Requirement of operation after CAP therapy was successfully predicted by using ANN. This newly established ANN strategy would be used as powerful support of physicians in the clinical practice.

## Introduction

Ulcerative colitis (UC) is a chronic and recurrent inflammatory disease of the colon. Most these patients are controlled with medication, but some are required total colectomy and frequent admissions. These remarkably disturb the quality of life (QOL) remarkably in many cases.

It is well known that UC is often associated with the increase of peripheral blood granulocytes and monocytes [1]. Accordingly, cytoapheresis therapy is widely used in UC patients with moderate to severe activity in Japan [2]. In this regard, several studies have shown the effect and safety of CAP [2–12]. Most of those studies showed that approximately 60% of patients have clinical response to CAP therapy [2–12].

Our previous study revealed that 1) long-term prognosis of UC patients who achieved remission by CAP therapy is favourable, 2) recurrent patients who previously showed efficacy with the first course of CAP successfully respond to the second course of CAP, and 3) the combination use of immunomodulators (IM) was effective to avoid operation and/or re-admission [13]. As the limitation of the study, we still could not have any suggestion which patients should be treated with IM.

According to the improvement of computer technology, computer-aided therapy is ready to be used in several areas of clinical practice [14–18]. If available computer-aided tool, which predict the outcome and/or prognosis of UC patients after CAP therapy with high accuracy from accumulating data, it is possible to prospectively simulate the patients by editing the each parameters before CAP therapy.

As for the system, we chose artificial neural network (ANN) system, which is a learning system based on a computational technique and has been previously used to simulate the neurological processing ability of the human brain [19]. ANN systems are widely used in various fields, such as the control of operation of the motor at constant speed, and air-conditioners’ controls. ANNs recognise complex patterns between inputs and outputs via the learning process. Once the hidden relationship between input and output has been learned, an ANN can correctly predict output from a given input [14, 15]. Notably, ANN systems use a non-linear expression in formulating the predictive value, therefore they do not suffer from the assessment of a non-linear relation [18]. Since recent study demonstrated that the kinetics of most phenomena in living organisms are non-linear [18], and ANNs can solve those relations, we chose ANNs for the generating the predictive expression. Recent reports showed the superiority of ANN to classical linear methods to solve several clinical problems such as in the prediction of effect of IFN-alpha and ribavirin combination therapy in patients with chronic hepatitis C infection [16, 17, 20–25], survival after percutaneous gastrostomy [26], incidence of metabolic syndrome [27], selection of lung cancer biomarkers [28], mortality risk in burn injury [29], diabetes complications [30] and post-operative bleeding risk [31]. The aims of this study are to develop a new tool that can support daily clinic by predicting the operation after CAP therapy by using clinical data and ANNs.

## Results

### Response rate and patient backgrounds

Ninety UC patients with moderate to severe activity (55 men and 35 women; mean age, 38·5 years; range of age, 14–77 years) received CAP therapy (Table 1). Of the 90 patients, 44 patients (48.8%) achieved remission, 14 patients (15.6%) had effect, and 32 patients (35.6%) had no effect. According to this result, we decided input and outcomes (Table 2). We divided the entire data in to training data set for generating predictive expressions and validation data set as detailed in method, generated the predictive expressions to predict the requirement of operation after CAP therapy from 13 input factors.

**Table 1:** Characteristics of patients.

	Total n = 90
Age (years)	38·5 ± 15·8 (14–77)
Gender	Male 55, Female 35
Type of CAP	GCAP 63, LCAP 27
Disease extent	Proctosigmoiditis 5, Left-sided colitis 26, Pancolitis 55, Others 4
Disease duration (years)	7·6 ± 7·1 (0–32)
Clinical Type	One-attack 6, relapsing-remitting 79, chronic continuous 5
CAI (before CAP)	9·2 ± 3·5
CAI (after CAP)	5·3 ± 4·1
Medication	
PSL	Yes 59, No 31
Immunomodurator (IM)	Yes 14, No 76
History of use of PSL	Yes 21, No 69
History of admission	Yes 20, No 70
History of operation	Yes 8, No 62

Continuous data are expressed as mean with range or number in parentheses. CAI: Clinical activity index, CAP: Cytoapheresis, PSL: Prednisolone

**Table 2:** Factors and outcome used to predict individual patient outcome.

Factors		Outcome
X_1_: Age	X_8_: CAI (after CAP)	Operation after CAP 0 = no, 1 = yes
X_2_: 1 = Male, 2 = Female	X_9_: Use of PSL before CAP 1 = yes, 2 = no	
X_3_: 1 = GCAP, 2 = LCAP	X_10:_ Use of 6-MP/AZA before CAP 1 = yes, 2 = no	
X_4_: Disease extent	X_11:_ History of admission	
X_5_: Duration (year)	X_12:_ History of PSL 1 = yes, 2 = no	
X_6_: Clinical type	X_13:_ History of operation 1 = yes, 2 = no	
X_7_: CAI (before CAP)		

### Sensitivity and specificity

To validate the predictive expressions, we analyzed the sensitivity and specificity. The sensitivity was 0.96 and the specificity was 0.97 (Table 3). Both sensitivity and specificity were considered high enough, suggesting that it can be used as support of physicians’ decision in daily clinic.

**Table 3:** The sensitivity and specificity provided by ANN.

Sensitivity	96%
Specificity	97%

### Past history of admission and operation non-linearly correlated to the outcome

**Fig 1:**
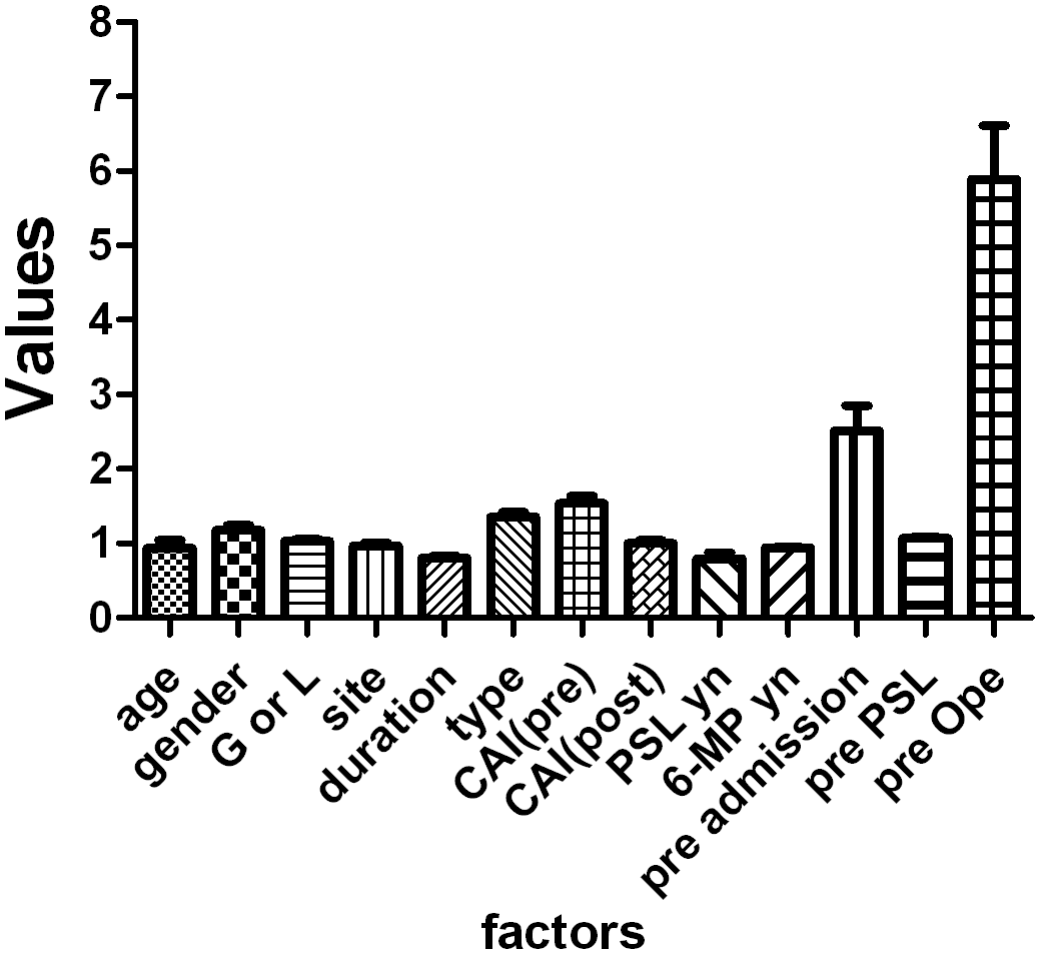
Relative weights of input factors for ANNs. Data are expressed as the mean±SD for member networks.

We next attempted to identify factors that critically correlate to the outcome in ANN by using the relative weights of input factors analysis (Fig 1 and S1 Table). This analysis involves determining how the result changes when the test factor (*X*_test_) is excluded. An *X*_test_ value greater than 1 indicates that it improves the expression, and a value less than 1 indicates that it does not improve the expression. We analysed all expressions and determined the corresponding means and standard deviations. As shown in Fig 1, *X*_13_ (history of operation) and *X*_11_ (history of admission) were defined as significant predictive factors in every trial.

### Independent four factors were the key factors to predict prospective operation

Given the evidence that events of operation and admission are significant as predictive factors, we deleted those two factors and generated the networks. As shown in Table 4, both sensitivity and specificity were decreased into 0.87 and 0.75 respectively by deleting those factors. Since we have previously reported the importance of use of IM and the effect of CAP to the requirement of operation after CAP therapy [13], we also assessed the predictive value without those 4 factors. Both sensitivity and specificity were dramatically decreased into 0.60 and 0.71, respectively, by deleting 4 factors, suggesting those 4 factors were critically related to the outcome.

**Table 4:** The sensitivity and specificity provided by ANN.

	All factors	Without 2 factors	Without 4 factors
Sensitivity	96%	87%	60%
Specificity	97%	75%	71%

## Discussion

In this study we successfully predicted the requirement of operation after CAP with high sensitivity and specificity (0.96 and 0.97 respectively). Since we use the non-linear analysis, we could not know the factor which correlate to outcome in linear way. However, using relative weights of input factors analysis, history of operation and history of admission were defined as important factors that influence to the prediction (Fig 1 and S1 Table). Moreover, gathering to our previous report, we defined two more factors which dramatically influence the outcome; use of IM and the effect of CAP.

In this study we set the simple factors as input. Moreover, use of IM were defined as important factor for the outcome, we can easily simulate and know the necessity of use of IM in individual patients from the simulated result.

Patients with UC suffer from repeated remissions and recurrences in refractory course. Especially, surgeon severely impairs the QOL. Accumulating reveal their pathology, new treatments and the effects, though, there are few reports, which show the prediction about prognosis. One of the difficulties in prediction of effect of treatment and/or prognosis is possibly existence of non-linear relation in several factors and the outcome. Most reports in clinical fields use classical linear approach, which could not solve non-linear relations. In contrast, ANNs can identify relationships within a patient’s clinical data that may be overlooked when classical linear approaches are used [18]. Because ANNs are trained using existing data, they are more capable of providing correct answers for individual patients. ANN can predict both linear and non-linear phenomena and can analyse relationships between many variables at different levels [23].

To avoid the bias as much as possible, in this study we randomly divided the all data into training data and validation data for 4 more times and evaluated the accuracy with same way. In all trials, both sensitivity and specificity were similar (S2 Table). We note that, although the ANN is a useful model, there is several limitations. First, the network logic of prediction cannot be broken down into simple elements because ANNs process data in a non-linear way [14, 18, 23, 32]. We used relative weights of input factors analysis to address this issue. Second, ANNs have over-fitting problems that occurs by convergence of expressions. The automatic ANN designer of the software we used avoid this issue by checking on progress of the algorithm using the independent selection set which randomly selected from entire data set.

Both physicians and patients express concern about the risks associated with treatment because it is difficult to predict the outcome at the time decisions are made. Since the increased demand for individualised treatment necessitates new statistics that can be applied in conjunction with ethical and clinical evidence at the individual level, ANNs may have potential economic benefits in that they reduce unnecessary medical treatment. Although our predictive expression does not predict responses completely, our results show that ANN is a valid method for devising individual treatment regimens in the clinical situation. It is well known that 100% prediction accuracy is impossible to achieve because of random error and multiple biases. Also we believe that it is very important to use various methods (including MLR, ANN and/or newly developed techniques [30]) to uncover the relation between multiple factors and multiple outcomes of diseases to realize the tailor-made medicine. As the outcome of CAP treatment and rate of operation may be affected by multiple unknown factors, it is important not only to update data continuously and to acquire clinical data such as the patient’s demographics, medical history, result of endoscopic examination, and laboratory test results, but also to demonstrate that the use of trained ANNs in routine medical practice increases the quality of medical care and reduces costs.

## Methods

### Patients

One hundred fourteen active UC patients who treated with CAP (granulocytapheresis; GCAP and/or leucocytaphereis; LCAP), and who could be followed-up more than 3 years were historically collected and data from 90 out of the patients were used for further analysis including background, medication and long-term prognosis. For long-term prognosis, we evaluated the rates of operation, re-admission, and use or dose-up of steroid. Clinical efficacy was evaluated by Clinical Activity Index (CAI).

This historical cohort study was conducted at the Keio university hospital. All patients’ data who underwent leukocyte apheresis for active UC from 2001 to 2006 were enrolled. 114 patients with clinically active ulcerative colitis treated with GCAP and/or LCAP once or twice a week. 90 patients’ data which could be followed the long time prognosis with full clinical data (55 men, 35 women; mean age, 36.4 years) were used for analysis. Average observation time was 4.59 years. Clinical efficacy was evaluated by Clinical Activity Index (CAI) according to the Rachmilewitz’s criteria with questionnaire to the patients [33]. We defined CAI less than 4 as remission, and more than 4 points decrease of CAI as effective according to previous reports [34]. This historical cohort study was conducted at Keio University Hospital and the study was approved by the Keio University School of Medicine review board and the permission was obtained. All patients who underwent CAP for UC with moderate to severe activity between 2001 and 2006 were enrolled. Written or oral informed consent was obtained from all patients and/or the parents of patients younger than 20 years of age.

A questionnaire was designed to review the demographic data, including age, gender, weight, height, either GCAP or LCAP, frequency of CAP therapy (once or twice per week), disease extent, duration, clinical type, CAI of pre 1^st^ course of CAP, and medication (use of PSL and/or IM). Outcomes (rates of operation, re-admission, and use or dose-up of steroid) were obtained from the hospital medical records.

### Inclusion criteria

Patients inclusion criteria: age between 14 and 77; had endoscopic and histologic diagnosis of UC, not indeterminate colitis; with colonic involvement, had a CAI score more than 5;

### Exclusion criteria

Exclusion criteria were patients with evidence of toxic megacolon; with malignancy, with serious concomitant cerebral, pulmonary, cardiac, hepatic or renal disease, and with a history of hypersensitivity reaction during an apheresis.

### Cyteapheresis therapy

Patients with moderately active disease were treated in our outpatient clinic, while those with severe disease were treated as in patients. Each patient received five or ten GCAP or LCAP once or twice per week. One GCAP session was 60min at 30ml/min and one LCAP session was 60min at 30–50ml/min. In patients who were receiving corticosteroids at entry, the dose of steroids was to be tapered or discontinued in line with clinical improvement during the CAP.

### ANN

To develop the ANN, we used three types of network according to manufacturer’s instruction: multilayer perceptrons (MLPs), radial-basis function networks (RBFs), and linear networks (LINs). Details of the ANN and MLP are provided elsewhere [35]. In brief, a hierarchical ANN consisting of three layers (one input, one hidden, and one output layer) was used to classify the effect as a node in the output layer. MLPs were constructed from three layers (one input, one hidden, and one output layer) to classify effects as a node in the output layer. RBF units respond to the distance of points from the centre. The RBF has a hidden layer of radial units, each of which models a Gaussian response surface. We analyzed the results of 90 patients from multiple centres and formed 100 000 networks.

### Training data set and validation data set

We randomly divided the entire dataset into a training dataset (n = 54, for generation of predictive expressions) and validation data set (n = 36). Validation data set was divided into selection set for internal validation and test set for external validation according to manufacturer’s instruction. We used same training data set for generating the predictive expression by using MLR and ANN, and used validation data set to evaluate the accuracy of the expression generated using training data set.

### Input factors and outcome

We used the clinical data to determine input factors *X*_1_–*X*_13_, which were used to predict the outcomes of individual patients using ANN analysis (Table 2). *X*_1_ and *X*_2_ represented the patient’s age and gender, respectively. *X*_3_ represented the type of CAP and *X*_4_–*X*_6_ represented the disease extent, duration and clinical type, respectively. *X*_7_ and *X*_8_ represented the CAI before and after CAP respectively. *X*_9_ and *X*_10_ represented medication (PSL and 6-MP/AZA, respectively). *X*_11_–*X*_13_ represented the history of admission, PSL, operation, respectively. The outcome was requirement of operation after CAP therapy.

### Relative weights of input factors analysis

The detail of relative weights of input factors analysis were described elsewhere [25, 36]. In brief, we analysed relative weights of input factors using a leave-one-input-factor-out (LOFO) in turn with a missing values substitution procedure, which enables predictions to be made in the absence of values for each causal factor, and then assessed effects upon ANN response error. Root mean square error (RMSE) is an estimate of the typical difference between the predicted and actual values of outcomes. The smaller RMSE is the better prediction accuracy of the models. The network original error was accumulated as RMSE_original_ and the network was again used with LOFO data and the error RMSE_LOFO_ was estimated. Then, the relative weights of input factors was calculated as RMSE_LOFO_/RMSE_original_.

### Data analysis

Multiple logistic analysis was performed using JMP version 7.0.1 software (SAS Institute Japan, Co., Ltd, Tokyo, Japan) and ANN was analysed using Statistica version 06J software (StatSoft Japan, Co., Ltd, Tokyo Japan).

## Supporting Information

S1 Table Values of relative weight of input factors analysis. (DOCX)

S2 Table Sensitivity and specificity of 4 other trials. (DOCX)
