# Supplementary material for: Computer-Aided Prediction of Long-Term Prognosis of Patients with Ulcerative Colitis after Cytoapheresis Therapy
Source: PLoS One. 2015 Jun 25;10(6):e0131197. doi: 10.1371/journal.pone.0131197 (PMC4481415; doi:10.1371/journal.pone.0131197)
Supplement: S1 Table — (DOCX) [file pone.0131197.s001.docx]

| **Factors** | **Values** |
| --- | --- |
| Age | 0.93 ± 0.23 |
| Gender | 1.18 ± 0.15 |
| GCAP or LCAP | 1.03 ± 0.05 |
| Site | 0.97 ± 0.09 |
| Duration | 0.80 ± 0.07 |
| Type | 1.36 ± 0.13 |
| CAI (pre CAP) | 1.54 ± 0.22 |
| CAI (post CAP) | 1.01 ± 0.08 |
| PSL | 0.79 ± 0.18 |
| 6-MP | 0.94 ± 0.04 |
| Pre admission | 2.51 ± 0.77 |
| Pre PSL | 1.07 ± 0.06 |
| Pre Operation | 5.89 ± 1.61 |
| Values show Average ± Standard deviation | |

**S1 Table. Values of relative weight of input factors analysis**
