# Supplementary material for: Computer-Aided Prediction of Long-Term Prognosis of Patients with Ulcerative Colitis after Cytoapheresis Therapy
Source: PLoS One. 2015 Jun 25;10(6):e0131197. doi: 10.1371/journal.pone.0131197 (PMC4481415; doi:10.1371/journal.pone.0131197)
Supplement: S2 Table — (DOCX) [file pone.0131197.s002.docx]

|  | Tiral 1 | Trial 2 | Trial 3 | Trial 4 |
| --- | --- | --- | --- | --- |
| Sensitivity (%) | 90 | 100 | 90 | 100 |
| Specificity (%) | 95 | 97 | 93 | 97 |

**S2 Table. Sensitivity and specificity of 4 other trials**
